# Detection and quantification of introgression using Bayesian inference based on conjugate priors

**DOI:** 10.1093/bioinformatics/btae642

**Published:** 2024-10-26

**Authors:** Bastian Pfeifer, Durrell D Kapan, Sereina A Herzog

**Affiliations:** Institute for Medical Informatics, Statistics and Documentation, Medical University Graz, Graz 8010, Austria; Department of Entomology and Center for Comparative Genomics, Institute for Biodiversity Science and Sustainability, California Academy of Sciences, San Francisco, CA 94118, United States; Institute for Medical Informatics, Statistics and Documentation, Medical University Graz, Graz 8010, Austria

## Abstract

**Summary:**

Introgression (the flow of genes between species) is a major force structuring the evolution of genomes, potentially providing raw material for adaptation. Here, we present a versatile Bayesian model selection approach for detecting and quantifying introgression, $d_{f-BF}$, that builds upon the recently published distance-based $d_{f}$ statistic. Unlike $d_{f}$, $d_{f-BF}$ accounts for the number of variant sites within a genomic region. The underlying model parameter of our $d_{f-BF}$ method, here denoted as $d_{f\theta }$, accurately quantifies introgression, and the corresponding Bayes Factors ($d_{f-BF}$) enables weighing the strength of evidence for introgression. To ensure fast computation, we use conjugate priors with no need for computationally demanding MCMC iterations. We compare our method with other approaches including $d_{f}$, $f_{d}$, $D_{p}$, and Patterson’s D using a wide range of coalescent simulations. Furthermore, we showcase the applicability of $d_{f-BF}$ and $d_{f\theta }$ using whole-genome mosquito data. Finally, we integrate the new method into the powerful genomics R-package PopGenome.

**Availability and implementation:**

The presented methods are implemented within the R-package PopGenome (https://github.com/pievos101/PopGenome) and the simulation as the application results can be reproduced from the source code available from a dedicated GitHub repository (https://github.com/pievos101/Introgression-Simulation).

## 1 Introduction

Hybridization among species is increasingly recognized as a pivotal evolutionary factor, challenging traditional views of genetic divergence and speciation (Whitney *et al.* 2006, Soltis and Soltis 2009, Kronforst *et al.* 2013, Harrison and Larson 2014). Contrary to the long-held view that species boundaries are impermeable, there is mounting evidence suggesting that they are semipermeable (Harrison and Larson 2014) and that resultant gene flow through hybridization plays a crucial role in shaping biodiversity and adaptation (Taylor and Larson 2019). This paradigm shift underscores the need for advanced methods to quantify hybridization and introgression—the transfer of genes between species (Hibbins and Hahn 2022).

The impacts of hybridization, gene flow, and introgression are multifaceted. Traditionally, hybridization was viewed negatively, with immediate fitness consequences for hybrid offspring (Whitlock *et al.* 2013, Adavoudi and Pilot 2021). Gene flow can decrease genetic differentiation, potentially leading to species loss through genomic swamping (Todesco *et al.* 2016). However, hybridization and gene flow can lead to introgression, where hybrids exchange genetic material with parental species, which can have negative or positive outcomes for recipient species (Aguillon *et al.* 2022). Introgression may lead to maladaptation and selection against introgressed DNA (Veller *et al.* 2023). Conversely, it could facilitate the transfer of adaptive alleles across species boundaries—a process known as adaptive introgression (Whitney *et al.* 2006, Hedrick 2013, Edelman and Mallet 2021). Paradoxically, introgression can also kick-start increased genetic differentiation between taxa (Edelman and Mallet 2021), creating novel gene combinations or introducing new genes into recipient genomic backgrounds (Barton 2001). Fitness-enhancing introgression can promote local adaptation (Hedrick 2013), facilitate range expansion (Pfennig *et al.* 2016), and help species respond to changing environments (Brauer *et al.* 2023). Ultimately, hybridization can contribute to species formation (Abbott *et al.* 2013) and lead to adaptive radiation (Seehausen 2004, Grant and Grant 2019, Meier *et al.* 2019).

Before the rise of relatively inexpensive genome-wide sequencing, much of what was known about hybrids was derived from studying phenotypically apparent hybrids found alongside parental species in the field, especially in showy species such as plants, some insects including Lepidoptera, and colorful birds (Mallet 2005). With the rise of next-generation genome sequencing, cases of hybridization and introgression are now widely documented across the entire tree of life (Taylor and Larson 2019, Edelman and Mallet 2021). Numerous tools have been developed to detect introgression at the genome scale, ranging from phylogenetic to population genetic approaches [reviewed in Hibbins and Hahn (2022)].

**Figure 1.**
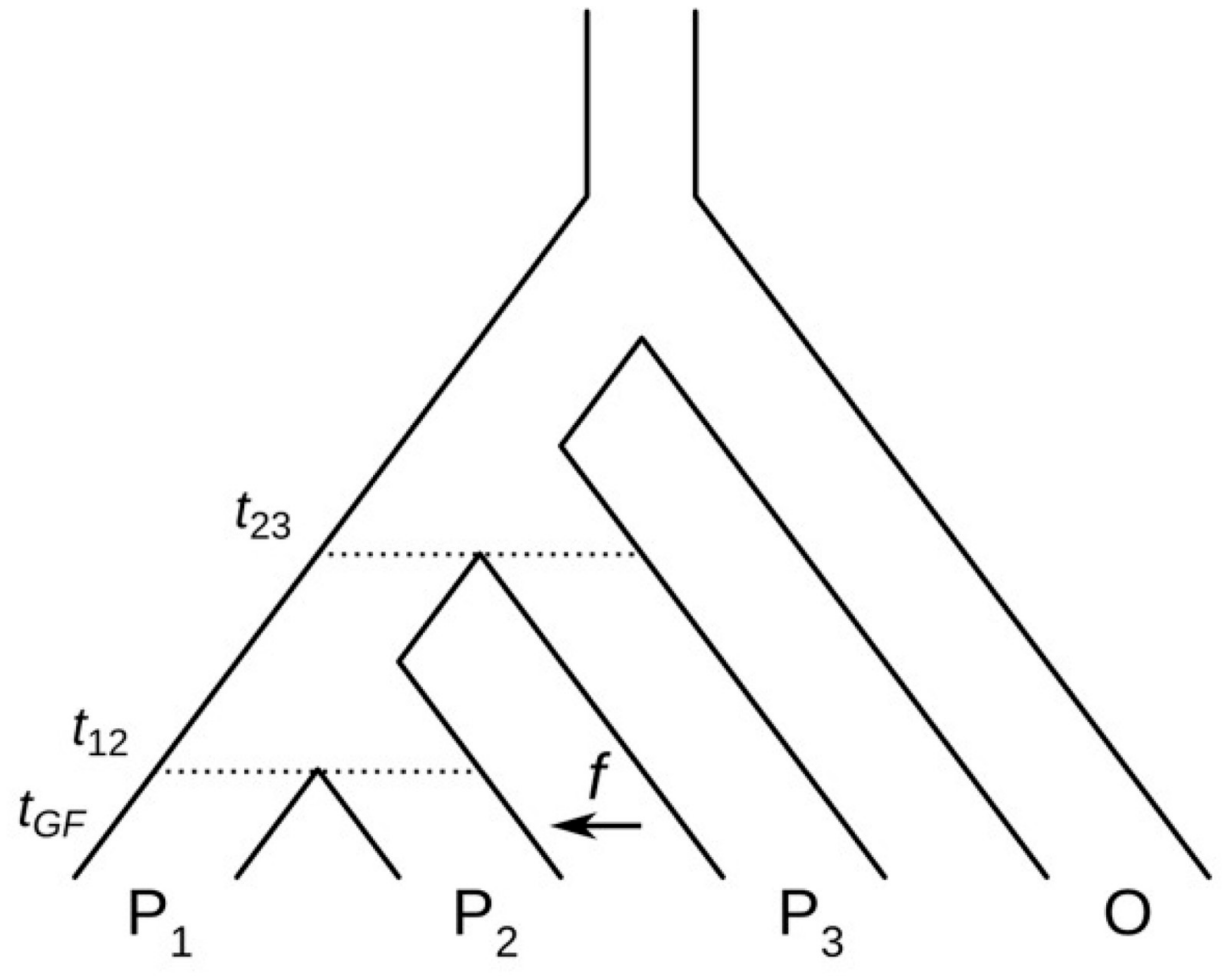
A four-taxon phylogenetic tree illustrating introgression between taxon 3 ($P_{3}$) and taxon 2 ($P_{2}$). The time of gene flow is indicated by $t_{GF}$, with the fraction of introgression *f*. The illustration is modified from Martin *et al*. (2015)

A unique combination of these two approaches derives from the four-taxon (see Fig. 1) test that compares sister species with a third species, and an outgroup (Kulathinal *et al.* 2009, Green *et al.* 2010). This method relies on an accounting of Single-Nucleotide Polymorphism (SNP) patterns arising from the sharing of a derived allele “B” between either member of the sister taxa with the third taxon (pattern “ABBA” indicates sharing between taxa P2 and P3, or “BABA” sharing between taxa P1 and P3). An excess of ABBA or BABA patterns is taken as evidence of introgression over the alternative of incomplete lineage sorting (Reich *et al.* 2010, Durand *et al.* 2011). Patterson’s D summarizes the imbalance of ABBA to BABA sites, indicating hybridization (Durand *et al.* 2011, Patterson *et al.* 2012).

However, it is now well understood that the D-statistic is biased, as it does not vary linearly with the fraction of introgression. Furthermore, when applied to smaller genomic regions, the D-statistic tends to overestimate the numbers of regions across the genome deemed introgressed, particularly in cases where reduced heterozygosity can lead to false positives (Martin *et al.* 2015).

Pfeifer and Kapan (2019) recognized that reduced genetic distance between taxa, an entirely obvious genomic signal of introgression, could be placed in the ABBA-BABA framework, to derive new measures of hybridization that did not suffer from the pitfalls of Patterson’s D. This paradigm shift underscores the need for advanced methods to quantify introgression in genomes (Hibbins and Hahn 2022), as understanding the dynamics of hybridization becomes imperative in unraveling the intricacies of evolutionary processes. Here, we extend the work of Pfeifer and Kapan (2019) by enhancing the $d_{f}$ distance statistic with Bayesian estimation.

We present a versatile Bayesian model selection approach for detecting and quantifying introgression, $d_{f-BF}$, which builds upon the distance-based $d_{f}$ statistic (Pfeifer and Kapan 2019). Unlike $d_{f}$, $d_{f-BF}$ accounts for the number of variant sites within a genomic region. The $d_{f-BF}$ method quantifies introgression with the inferred θ parameter and simultaneously enables weighing the strength of evidence for introgression based on Bayes Factors. We employ conjugate priors, which eliminate the need for computationally demanding MCMC iterations to ensure fast computation.

The article is structured as follows: Section 2 briefly reviews the $d_{f}$ statistic and Section 3 introduces our new Bayesian approach. We evaluate this approach through simulations based on previous research on introgression levels and numeric experiments involving maximally introgressed SNPs. This allows us to emphasize the impact of population size and the genomic scale of measurement. The corresponding simulation setup is detailed in Section 4. Results from these simulations are presented in Section 5, which also includes an application to real-world data.

## 2 The $d_{f}$ statistic

The $d_{f}$ statistic (Pfeifer and Kapan 2019), an estimator of the proportion of introgression, is formulated as

(1) $$d_{f}=\frac{\sum_{k}^{L}\left(\mathrm{ABB}A_{k}-\mathrm{BAB}A_{k}\right)}{\sum_{k}^{L}\left(\mathrm{ABB}A_{k}+\mathrm{BAB}A_{k}+2\cdot \mathrm{BBA}A_{k}\right)},$$

where $\mathrm{ABB}A_{k}$, $\mathrm{BAB}A_{k}$, and $\mathrm{BBA}A_{k}$ represent SNP sharing patterns on a four-taxon tree, which we show can be expressed in terms of genetic distance:

(2) $$d_{f}=\frac{\sum_{k}^{L}\left(p_{2k}\cdot d_{13k}-p_{1k}\cdot d_{23k}\right)}{\sum_{k}^{L}\left(p_{2k}\cdot d_{13k}+p_{1k}\cdot d_{23k}\right)},$$

where $p_{xk}$ refers to the mutant allele frequency in population x at variant site k. Here, $d_{\mathrm{xyk}}$ is the average pairwise nucleotide difference between population x and population y at variant site k. L is the total number of bi-allelic sites in a genomic region. The first two taxa are closely related species the third taxon is a potential donor of mutant allele B at variable sites, and the fourth taxon refers to the outgroup as in the original work by Patterson (Green *et al.* 2010). Note, that the $d_{f}$ statistic calculates the fraction of introgression based on variant sites where the outgroup (taxon 4) is monomorphic for allele A. From Equation (2), it can be seen that when either $p_{2k}\cdot d_{13k}$ = $(\mathrm{ABB}A_{k}+\mathrm{BBA}A_{k}$) or $p_{1k}\cdot d_{23k}$ = $(\mathrm{BAB}A_{k}+\mathrm{BBA}A_{k}$) is zero, the $d_{f}$ statistic estimate is 1 or −1, respectively. This can generate false positives in low diversity regions, e.g. in low recombining regions comprising only a few bi-allelic markers. This issue is not unique to the $d_{f}$ statistic, it applies to other *ABBA*−*BABA* methods, such as $f_{d}$ (Martin *et al.* 2015), and Patterson’s D since they do not explicitly account for the number of bi-allelic sites.

To tackle this problem, we transform the $d_{f}$ statistic into a Bayesian model selection problem.

## 3 New approach

We define two competing models of introgression.

$$\begin{array}{c} M_{\mathrm{ABBA}}:\text{Taxa}\;2\;\&\;3\;\text{are}\;\text{sharing}\;\text{alleles}\;(P_{2}↔P_{3})\\ M_{\mathrm{BABA}}:\text{Taxa}\;1\;\&\;3\;\text{are}\;\text{sharing}\;\text{alleles}\;(P_{1}↔P_{3}) \end{array}.$$

Models $M_{\mathrm{ABBA}}$ and $M_{\mathrm{BABA}}$ can be conceptually represented by the following likelihood functions for the observed data D:

(3) $$Pr(D|M_{\mathrm{ABBA}})\propto \theta_{1}^{\alpha_{\mathrm{ABBA}}}\cdot \theta_{2}^{\beta_{\mathrm{BBAA}}}.$$

(4) $$\begin{array}{c} Pr(D|M_{\mathrm{BABA}})\propto \theta_{1}^{\alpha_{\mathrm{BABA}}}\cdot \theta_{2}^{\beta_{\mathrm{BBAA}}}. \end{array}$$

The parameter $\theta_{1}$ in model $M_{\mathrm{ABBA}}$ includes information about the fraction of the data explained by the ABBA+BBAA ($\alpha_{\mathrm{ABBA}}=p_{2}\cdot d_{13}$) patterns. In model $M_{\mathrm{BABA}}$, $\theta_{1}$ captures the BABA+BBAA ($\alpha_{\mathrm{BABA}}=p_{1}\cdot d_{23}$) signals. The parameter $\theta_{2}$ includes the species tree pattern BBAA ($\beta_{\mathrm{BBAA}}=p_{1}\cdot p_{2}\cdot (1-p_{3})$), which is used as an approximate measure of the neutral (non-introgressed) signal within the data (for more details, see Pfeifer and Kapan 2019). We perform Bayesian inference over the parameter space of conjugate Beta distributions with $\theta_{2}=1-\theta_{1}$, which assumes a process *proportional* to a binomial likelihood [see Equations (3) and (4)]. The shape parameters are $\alpha_{\mathrm{ABBA}}$, $\alpha_{\mathrm{BABA}}$, and $\beta_{\mathrm{BBAA}}$, computed from the data D using the $d_{f}$ logic. The expected value of the computed Beta posterior distribution is our new estimate of introgression, here denoted as $d_{f\theta }$.

The Bayesian model assumes that the observed data D can be approximately explained by the species tree pattern (BBAA) plus the corresponding introgression frequency patterns (ABBA and BABA). We use the following conjugate Beta distribution B as a prior

(5) $$Pr(M_{\mathrm{ABBA}})=B(\alpha_{\mathrm{ABBA}}=\lambda_{p},\beta_{\mathrm{BBAA}}=\lambda_{p}),$$

(6) $$Pr(M_{\mathrm{BABA}})=B(\alpha_{\mathrm{BABA}}=\lambda_{p},\beta_{\mathrm{BBAA}}=\lambda_{p}),$$

where $\lambda_{p}$ is the initial guess for *no* introgression evidence, adjusting the sensitivity to the actual observed signals of introgression in the data. To form the posterior, we use the following updating scheme of the Beta distribution per variant site k

(7) $$\alpha_{\mathrm{ABBA}}=\lambda_{s}\cdot \sum_{k}^{L}p_{2k}\cdot d_{13k},$$

(8) $$\alpha_{\mathrm{BABA}}=\lambda_{s}\cdot \sum_{k}^{L}p_{1k}\cdot d_{23k},$$

(9) $$\beta_{\mathrm{BBAA}}=\lambda_{s}\cdot \sum_{k}^{L}p_{1k}\cdot p_{2k}\cdot (1-p_{3k}),$$

where $\lambda_{s}$ is a scaling factor that we set to the average population size of $P_{1}$, $P_{2}$, and $P_{3}$. The corresponding posterior density distributions of the models $M_{\mathrm{ABBA}}$ and $M_{\mathrm{BABA}}$ are

(10) $$L(D|M_{\mathrm{ABBA}},d_{f\theta })\propto \log\frac{\Gamma (\lambda_{p}+\alpha_{\mathrm{ABBA}})\cdot \Gamma (\lambda_{p}+\beta_{\mathrm{BBAA}})}{\Gamma (\alpha_{\mathrm{ABBA}}+\beta_{\mathrm{BBAA}}+2\cdot \lambda_{p})},$$

(11) $$\begin{array}{c} L(D|M_{\mathrm{BABA}},d_{f\theta })\propto \log\frac{\Gamma (\lambda_{p}+\alpha_{\mathrm{BABA}})\cdot \Gamma (\lambda_{p}+\beta_{\mathrm{BBAA}})}{\Gamma (\alpha_{\mathrm{BABA}}+\beta_{\mathrm{BBAA}}+2\cdot \lambda_{p})}, \end{array}$$

where $d_{f\theta }$ is the inferred Beta model parameter to quantify the gene flow between $P_{2}↔P_{3}$ (model $M_{\mathrm{ABBA}}$) and $P_{1}↔P_{3}$ (model $M_{\mathrm{BABA}}$). Finally, evidence of introgression is calculated using Bayes Factors as

(12) $$\begin{array}{c} d_{f-BF}=\{\begin{array}{cc} 1, & d_{f}=0\\ \;\text{exp}(\frac{L(D|M_{\mathrm{ABBA}},d_{f\theta })}{L(D|M_{\mathrm{BABA}},d_{f\theta })})-\text{exp}(1)+1, & d_{f}>0\\ \;\text{exp}(\frac{L(D|M_{\mathrm{BABA}},d_{f\theta })}{L(D|M_{\mathrm{ABBA}},d_{f\theta })})-\text{exp}(1)+1, & d_{f}<0 \end{array} \end{array},$$

allowing researchers to judge the relative merit of the two competing introgression models. We scaled the obtained likelihood fractions in Equation (12) using the exponential function exp() so that the resulting Bayes Factors can be interpreted according to Jeffreys (Jeffreys 1939). See Table 1 for an overview of these values.

**Table 1.** Jeffreys’ scale for Bayes Factors interpretation.

Bayes Factor (BF)	Strength of evidence
1	No evidence
(1,3]	Anecdotal evidence
(3,10]	Moderate evidence
(10,30]	Strong evidence
(30,100]	Very strong evidence
>100	Extreme evidence

It should be noted again that the BBAA pattern is included in both $\theta_{1}$ and $\theta_{2}$ [see Equations (3) and (4)]. This is true for both models, $M_{\mathrm{ABBA}}$ and $M_{\mathrm{BABA}}$, and thus we are performing inference on the [0.5,1] theta range. Therefore, a transformation back into the full theta range [0,1] is required to estimate the fraction of introgression. We define $d_{f\theta }$ as

(13) $$\begin{array}{c} d_{f\theta }=\{\begin{array}{cc} 0, & d_{f}=0\\ \alpha_{\mathrm{ABBA}}/(\alpha_{\mathrm{ABBA}}+\beta_{\mathrm{BBAA}})/0.5-1, & d_{f}>0\\ \alpha_{\mathrm{BABA}}/(\alpha_{\mathrm{BABA}}+\beta_{\mathrm{BBAA}})/0.5-1, & d_{f}<0 \end{array} \end{array}.$$

In our software implementation, the parameter $d_{f\theta }$ can optionally be set to negative values when $d_{f}<0$ (indicating introgression between $P_{3}$ and $P_{1}$, model $M_{\mathrm{BABA}}$ supported), while it remains positive when $d_{f}>0$ (indicating introgression between $P_{3}$ and $P_{2}$, model $M_{\mathrm{ABBA}}$ supported). This flexibility allows the user to reflect the most highly supported model of introgression based on the combination of $d_{f\theta }$ and the posterior support for the introgression model, which is especially useful when graphing the results of the calculations.

Given the described mathematical model we can quantify the fraction of introgression and at the same time verify the supporting strength of the signal using Bayes Factors.

## 4 Simulation set-up

To validate our approach, we follow our previous simulation setup (Pfeifer and Kapan 2019) built upon that presented by Martin *et al.* (2015).

First, we generated topologies with different levels of introgression using Hudson’s **ms** program (Hudson 2002). It is extensively utilized for simulating genetic variation data, specifically SNP data, by randomly sampling haplotypes from a population. Users can customize several parameters related to population demography, such as population sizes and migration patterns, as well as evolutionary factors like mutation, crossover, and gene conversion rates.

Second, the sequence alignments were produced by the **seq-gen** program (Rambaut and Grass 1997). Seq-Gen is a versatile program designed to simulate the evolution of nucleotide or amino acid sequences along a phylogeny, employing various substitution models, including the general reversible model. Users can specify parameters like state frequencies and incorporate site-specific rate heterogeneity in multiple ways. The program accommodates the input of multiple trees, generating numerous datasets for each tree, making it suitable for creating extensive sets of replicate simulations. Overall, Seq-Gen serves as a general-purpose simulator, encompassing commonly used and computationally tractable models of molecular sequence evolution.

We generated 5 kb sequences with split times $t_{12}=1\times 4N$, $t_{123}=2\times 4N$, and $t_{123O}=3\times 4N$ generations ago. The time of gene flow from $P_{3}$ to $P_{2}$ was set to $t_{GF}=0.1\times 4N$ generations ago with a fraction of introgression of f=0.1. The recombination rate was set to r=0.01, and the Hasegawa–Kishino–Yano substitution model was employed, utilizing a branch scaling factor of s=0.01. We varied the fraction of introgression f and the time of gene flow $t_{GF}$. For each set-up, we repeated the simulation 100 times and for each run we computed $d_{f\theta }$, Patterson’s D (Patterson *et al.* 2012), $f_{d}$ (Martin *et al.* 2015), $D_{p}$ (Hamlin *et al.* 2020), and $d_{f}$ (Pfeifer and Kapan 2019). Detailed guidance for generating synthetic data using **ms** and **seq-gen** is provided on our GitHub repository (https://github.com/pievos101/Introgression-Simulation).

In addition to the above-described simulation, to study how $d_{f-BF}$ values are affected by changes in population size, the prior $\lambda_{p}$, and the number of SNPs supporting the simulated signal, we modeled a single SNP with maximal possible introgression (f=1) from $P_{3}$ to $P_{2}$. We set minor allele frequencies to $p_{1}=0$, $p_{2}=1$, and $p_{3}=1$ and proceeded to study these effects based on this single marker and copies of that marker.

## 5 Results and discussion

### 5.1 Simulations

**Figure 2.**
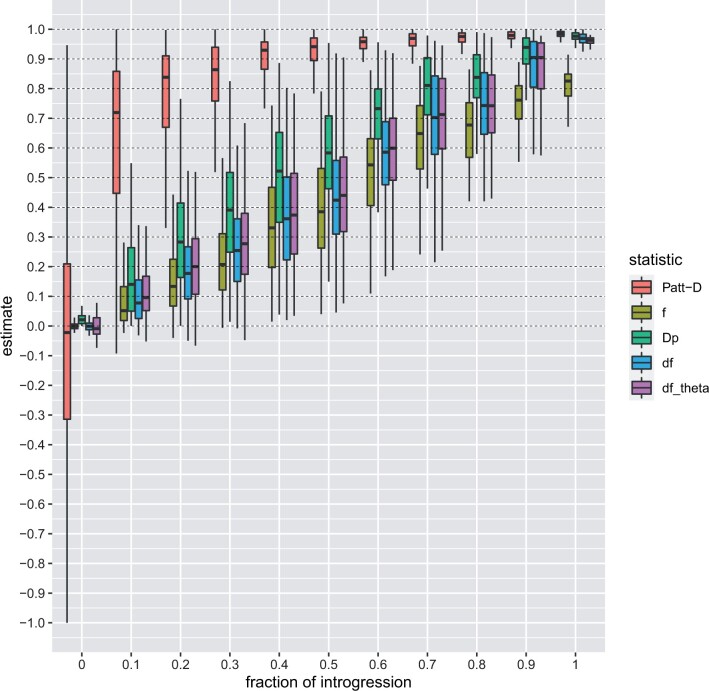
Simulation results—the fraction of introgression. Results comparing estimators Patterson’s *D*, $f_{d}$, $D_{p}$, $d_{f}$, and $d_{f\theta }$ for varying levels of introgressionf(f∈{0,0.1,…,1};100 iterations each). The horizontal dashed lines refer to the real fraction of introgression

**Figure 3.**
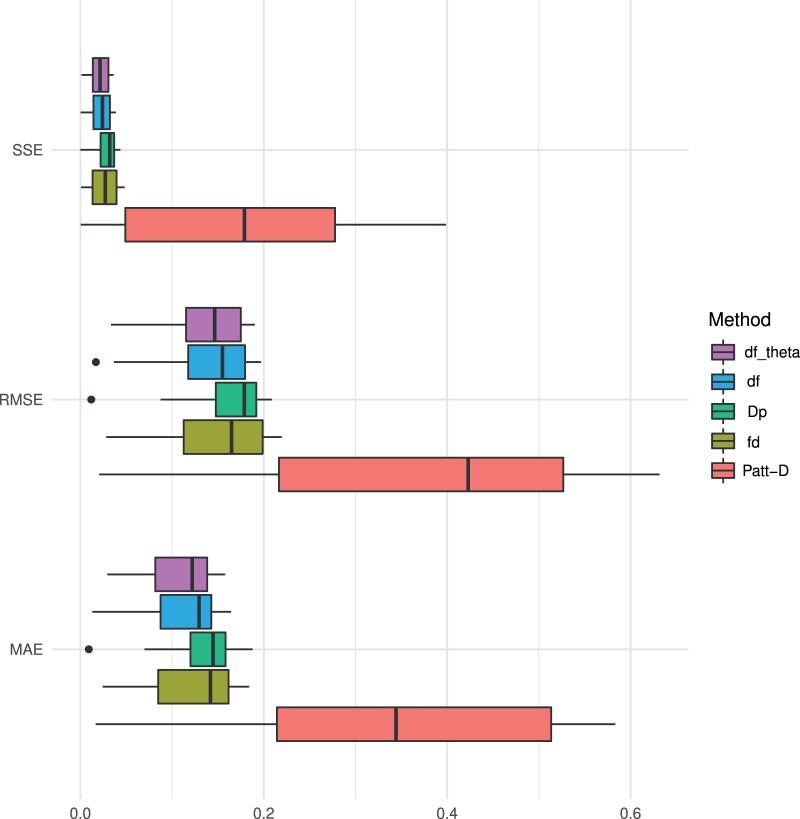
Simulation results—the fraction of introgression. The metrics Sum of Squared Errors (SSE), Root Mean Squared Error (RMSE), and Mean Absolute Error (MAE) values of the statistics Patterson’s *D*, $f_{d}$, $D_{p}$, $d_{f}$, and $d_{f\theta }$ (bottom to top) over *all* simulated data with varying levels of introgression f(f∈{0,0.1,…,1};100 iterations each)

The results based on synthetic data show that $d_{f\theta }$ reliably estimates introgression and $d_{f-BF}$ quantifies the evidence of introgression versus the null model. Figure 2 displays the results of our experiments when varying the fraction of introgression from population $P_{3}$ to $P_{2}$. The $d_{f-BF}$ model parameter θ (denoted as $d_{f\theta }$) precisely quantifies the fraction of introgression and produces almost identical results as the $d_{f}$ statistic (see Fig. 2). Patterson’s D greatly overestimates the fraction of introgression on the whole spectrum. The $f_{d}$ method tends to underestimate the fraction of introgression, especially when introgression is strong (Fig. 2). In Figure 3, we report on the Sum of Squared Errors (SSE), Root Mean Squared Error (RMSE), and Mean Absolute Error (MAE). The $d_{f\theta }$ model parameter consistently outperforms the competing methods for all metrics when the median is used for comparison. These results indicate that $d_{f\theta }$ performs best across the spectrum of possible levels of introgression.

**Figure 4.**
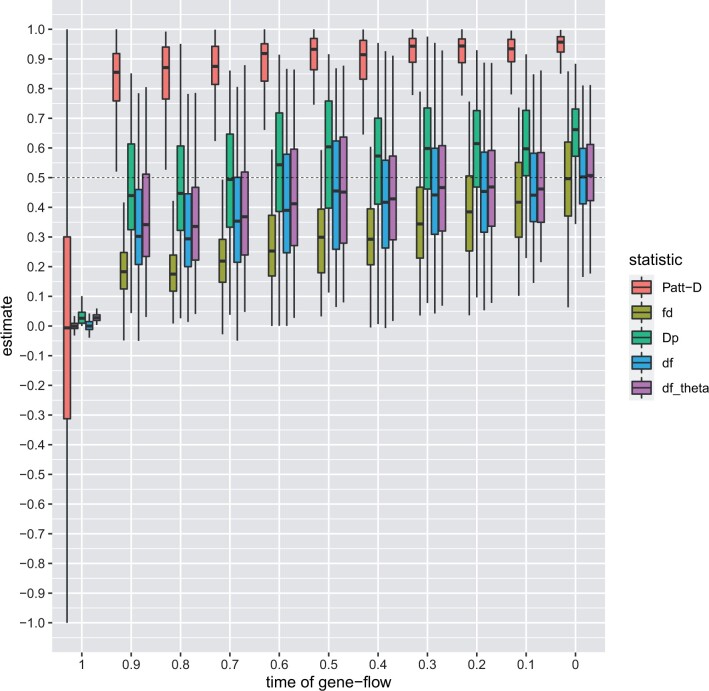
Simulation results—time of gene-flow. Results of the statistics Patterson’s *D*, $f_{d}$, $D_{p}$, $d_{f}$, and $d_{f\theta }$ on simulated data with varying time of gene-flow $t_{GF}$ ($t_{GF}\in \{0,0.1,\ldots ,1\};100\;\text{iterations}\;\text{each}$). The horizontal dashed line refers to the real simulated fraction of introgression (f=0.5)

**Figure 5.**
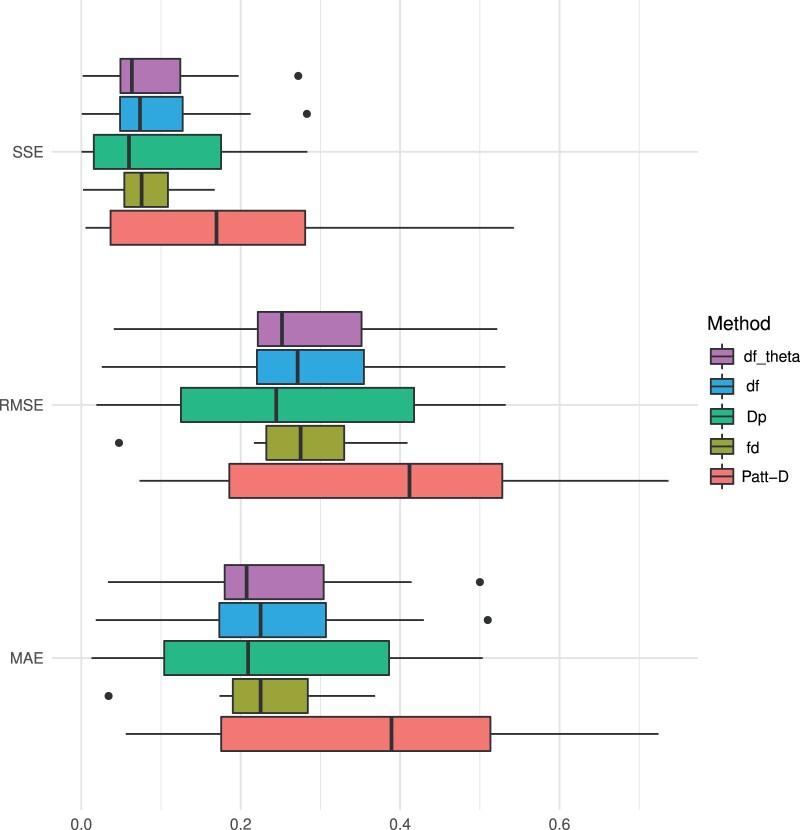
Simulation results—time of gene flow. The metrics Sum of Squared Errors (SSE), Root Mean Squared Error (RMSE), and Mean Absolute Error (MAE) values of the statistics Patterson’s *D*, $f_{d}$, $D_{p}$, $d_{f}$, and $d_{f\theta }$ (bottom to top) over *all* simulated data with varying the time of gene-flow $t_{GF}$ ($t_{GF}\in \{0,0.1,\ldots ,1\};100\;\text{iterations}\;\text{each}$). The fraction of introgression *f* is set to 0.5

We also varied the time of gene flow. We could confirm the results reported by Pfeifer and Kapan (2019); $d_{f}$ is nearly unaffected by the time of gene flow, and quantifies the fraction of introgression more accurately compared to Patterson’s D and $f_{d}$. Here, we report the same properties for $d_{f\theta }$ (see Fig. 4). When varying the time of gene flow (Figs 4 and 5) $d_{f\theta }$ and $D_{p}$ are the best-performing estimates of introgression. $D_{p}$ is more accurate for low levels of introgression, while $d_{f\theta }$ is a much better estimator when the signal of gene flow is strong. Furthermore, Fig. 4 shows that $f_{d}$ strongly underestimates the introgression. Again, the values based on Patterson’s D are inflated.

Introgression statistics, including $d_{f}$, are influenced by evolutionary factors such as branch lengths and effective population sizes, as demonstrated in Pfeifer and Kapan (2019). Further development utilizing the Bayesian framework introduced here offers promise to address these additional evolutionary effects.

**Figure 6.**
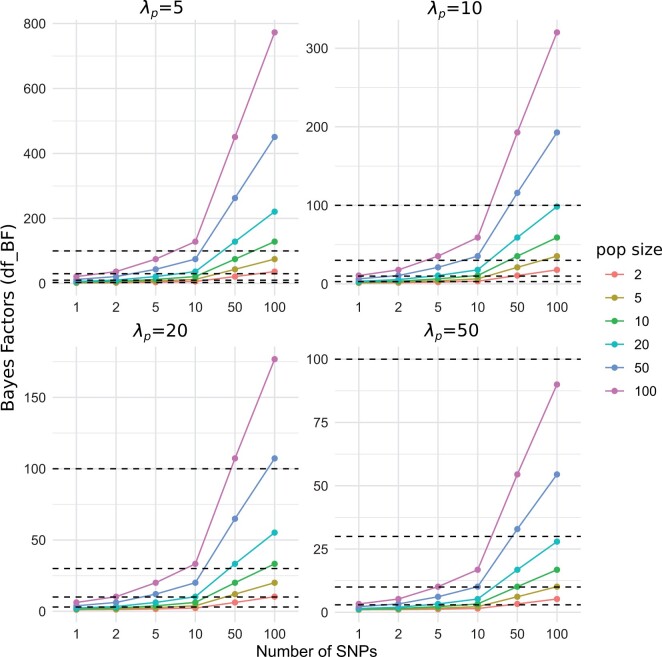
Change in $d_{f-BF}$ values as a function of varying the average population size ($\lambda_{s}$), priors ($\lambda_{p}$), and the number of SNPs supporting the signal of introgression. The fraction of introgression is f=1 for each set-up. The horizontal dashed lines refer to the critical values according to Jeffrey’s Table (see Table 1)

Moreover, we extensively examined the scaling characteristics of the Bayes Factors. We simulated maximal possible introgression (f=1), varied the number of SNPs within a genomic region supporting that signal, and investigated the influence of the population size accordingly. Figure 6 reveals the scaling of the $d_{f-BF}$ values are dependent on the prior $Pr(M_{\mathrm{ABBA}})$, $Pr(M_{\mathrm{ABBA}})$, and the corresponding $\lambda_{p}$ values. When $\lambda_{p}$ is set to the average population size ($\lambda_{p}=\lambda_{s})$) it results in an overly conservative test. With this specific setting, very-strong evidence of introgression ($d_{f-BF}>30$) is obtained with 50 consecutive SNPs supporting complete introgression. With $\lambda_{p}=5$ this critical value is already reached with 10 SNPs. The prior-specific $\lambda_{p}$ values can be adjusted by the user as a flexible parameter within our provided software implementation, with Fig. 6 providing guidance. For example, with an average population size of 100, a researcher might view 10 consecutive SNPs under complete introgression as a very strong indication of introgression ($d_{f-BF}\in (30,100]$). Figure 6 demonstrates that in this scenario, strong evidence is achieved using a prior-specific $\lambda_{p}=10$, as indicated in the upper right panel.

**Figure 7.**
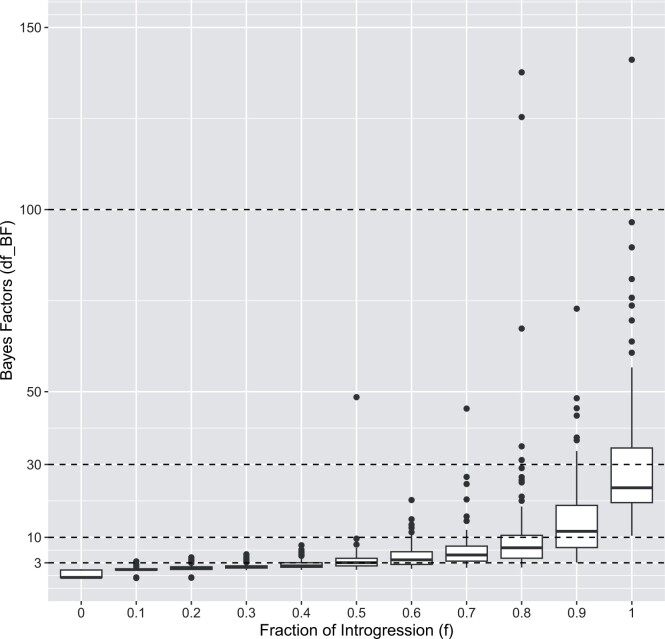
Bayes Factors ($d_{f-BF}$) for simulated data with varying levels of introgressionf(f∈{0,0.1,…,1};100 iterations each). The horizontal dashed lines refer to the critical values according to Jeffrey’s Table (see Table 1). The displayed Bayes Factors correspond to the simulated data shown in Fig. 2

The displayed Bayes Factors in Fig. 7 correspond to the simulated data shown in Fig. 2. With the current default parameter setting strong evidence of introgression is reported when the fraction of introgression is >0.8.

### 5.2 Application

We applied the $d_{f-BF}$ method to mosquito data analyzed by Fontaine *et al.* (2015). Their investigation identified introgression between the species *Anopheles merus* and *Anopheles quadriannulatus*. Chromoplots for all five chromosomal arms showed a highly spatially heterogeneous distribution of phylogenies, particularly on 3L (Fontaine *et al.* 2015). The authors studied three possible rooted phylogenetic relationships for *An. quadriannulatus* (Q), *An. melas* (L), and *An. merus* (R), with *An. christyi* as an outgroup. The region on 3L showed strong evidence of R-Q introgression and a strong negative deviation of the Patterson’s D statistic.

The average population size of the studied groups was 46. Therefore, we set $\lambda_{p}=15$, which provides strong evidence of introgression ($d_{f-BF}\in (10,30]$) when there are approximately 10 fully introgressed SNPs (see Fig. 6).

**Figure 8.**
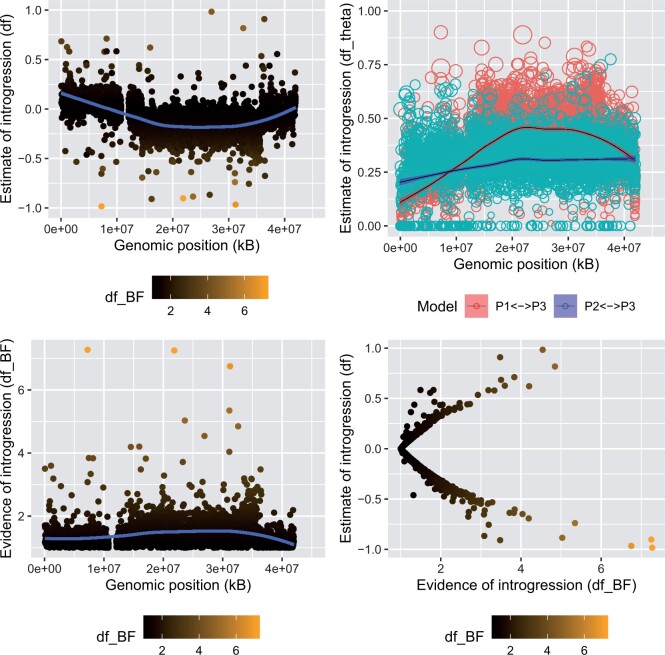
Application for the 3La chromosome of *Anopheles gambiae*. The chromosome was scanned using 10-kB consecutive windows. Values for $d_{f}$ (upper left) vs $d_{f\theta }$ colored by each alternative specified introgression model where the size of the circle is proportional to $d_{f-BF}$ (upper right), $d_{f-BF}$ (lower left), and the latter vs $d_{f}$ on the lower right. Note, points are colored by $d_{f-BF}$ (scale on bottom)

Next, we scanned the 3L arm with 10-kb consecutive sliding windows and found $d_{f\theta }$ detects introgression (Fig. 8). The panel in the bottom right plots the Bayes Factors and the $d_{f}$ statistic values for the same genomic regions. The plot shows that $d_{f}$ false positives are resolved by $d_{f-BF}$. In extreme cases, $d_{f}$ assigns a maximal fraction of introgression to a region, while the $d_{f-BF}$ Bayes Factor reports no evidence of introgression. This is because, in these regions, the estimated fraction of introgression is supported by only a few variant sites, or in extreme cases, just a single SNP. There are three genomic regions where *moderate* evidence of introgression can be reported ($d_{f-BF}>6$). The detected genomic regions differ in their estimated value of the fraction of introgression, the evidence of introgression, and the number of supporting SNPs (see Table 2). For instance, while the window at 7 Mb has the highest $d_{f}$ value, the evidence of introgression measured by $d_{f-BF}$ is almost identical to the genomic window at 21 Mb. This is because, the 7 Mb window contains only two SNPs, both with strong signals of introgression, whereas within the genomic region at 21 Mb, there are multiple SNPs. Not all of them contain a strong signal; otherwise, the corresponding $d_{f-BF}$ value would be much higher (see Fig. 6).

**Table 2.** Detected regions on the *Anopheles gambiae* 3La chromosome.

Mb (start)	Mb (end)	Number of SNPs	$d_{f}$	$d_{f-BF}$
7.26	7.27	2	−0.98	7.27
21.85	21.86	10	−0.90	7.25
31.23	31.24	3	−0.97	6.75

Overall, the signal of the introgressed vector is moderate but consistent over multiple consecutive windows.

### 5.3 Implementation

The $d_{f\theta }$/$d_{Bf}$ methodology is fully integrated into the widely used R-package PopGenome (Pfeifer and Kapan 2019) and can scan across genomes using narrow sliding windows, chromosomes, or whole genomes. These methods are found in a dedicated PopGenome module, called introgression.stats(). By integrating the presented methods into PopGenome, researchers can use the full functional spectrum of PopGenome, significantly simplifying data analysis. To reproduce the results shown in Fig. 8, we provide source code on our GitHub repository (https://github.com/pievos101/Introgression-Simulation).

## 6 Conclusion

We have developed a versatile Bayesian model selection framework that effectively detects and quantifies introgression, exhibiting accuracy equal to or surpassing that of the $d_{f}$ statistic upon which it is founded. Concurrently, it allows for quantification of the estimated value and the corresponding strength of evidence for introgression through the utilization of Bayes Factors. We have incorporated the new method into the robust genomics R-package PopGenome, which is readily accessible on GitHub (https://github.com/pievos101/PopGenome).

## Data Availability

The presented methods are implemented within the R-package PopGenome (https://github.com/pievos101/PopGenome) and the simulation can be reproduced from the code available on a dedicated GitHub repository (https://github.com/pievos101/Introgression-Simulation). The mosquito data are available on Dryad (https://datadryad.org/stash/dataset/doi:10.5061/dryad.f4114).
